# Bibliometric Analysis of Classroom Engagement: A Review Based on Web of Science Database

**DOI:** 10.3390/bs15060737

**Published:** 2025-05-26

**Authors:** Zhen Zhang, Yali Zhao, Xiaoyu Huang, Chunhui Qi, Guoxiang Zhao

**Affiliations:** 1 Faculty of Education, Henan Normal University, Xinxiang 453007, China; zhangzhenpsy@126.com (Z.Z.); 18738389210@163.com (Y.Z.); 15516440900@163.com (X.H.); 2 Faculty of Education, Henan University, Kaifeng 475001, China

**Keywords:** classroom engagement, visualization analysis, knowledge map, CiteSpace, Web of Science

## Abstract

Classroom engagement, a critical factor in enhancing learning outcomes and personal development, serves as a direct manifestation of students’ agency in learning. Understanding its developmental trajectory facilitates the efficient implementation of pedagogical activities. It also promotes students’ holistic development. This study aims to delineate the dynamic evolution of classroom engagement by constructing a network-based knowledge map, thereby revealing overarching research trends and shifts in this field. Systematically reviewing literature on classroom engagement since 1975, this research employs CiteSpace to visualize 919 articles sourced from the Web of Science Core Collection, offering valuable insights for theoretical exploration and practical applications in this domain. Key findings indicate: (1) a consistent increase in classroom engagement research over the past five decades; (2) the United States as the leading contributor; (3) Arizona State University, Texas A&M University College Station, and the University of California System as the most prolific institutions; (4) Fitzpatrick C as the most representative high-output author, with Fredricks JA being the most frequently cited scholar; (5) core journals including *Journal of Educational Psychology*, *Review of Educational Research*, and *Child Development*; and (6) emerging research hotspots such as flipped classroom, language, online education, and three focal themes: students with disabilities, interpersonal relationships, and student engagement.

## 1. Introduction

High-quality instruction remains fundamental to educational success, exerting significant influence not only on students’ personal growth but also on broader educational systems, societal advancement, and national development (Lai & Peng, 2019). As the primary setting for educational interactions, classroom teaching constitutes students’ most frequent and impactful learning experience (Abdullah et al., 2012). Substantial research indicates that students’ classroom engagement significantly affects both psychological development and academic outcomes, while serving as a critical mechanism for mitigating prevalent educational challenges including academic underperformance, disengagement, and attrition (Hardre & Reeve, 2003; Jang et al., 2009; Sereno et al., 2020; M. T. Wang & Fredricks, 2014). In student-centered classrooms, classroom engagement serves not only as a crucial indicator of instructional effectiveness but also as a pivotal metric for assessing student performance (Virtanen et al., 2015). Current research has revealed two distinct approaches: some scholars investigate generalized school engagement encompassing academic activities, extracurricular practices, and social interactions (Fredricks et al., 2004), while others concentrate specifically on classroom settings to examine students’ deep behavioral and cognitive involvement in learning processes (Skinner et al., 2009; M. T. Wang & Eccles, 2013). Given the stronger conceptual alignment between classroom engagement and academic development compared to general school engagement (Martins et al., 2022), this study systematically analyzes classroom learning behaviors to elucidate the mechanisms influencing engagement patterns within specific instructional contexts.

The conceptualization of student engagement has evolved from unidimensional constructs to multidimensional frameworks as its core significance gained academic recognition. Early researchers, drawing on observational and practical experience, deconstructed engagement into two foundational dimensions: behavioral and emotional (Finn, 1993; Marks, 2000). Subsequently, Fredricks et al.’s (2004) tripartite model achieved broad acceptance, defining school engagement as a multifaceted concept encompassing interrelated behavioral, emotional, and cognitive dimensions. Behavioral engagement manifests through students’ involvement in academic, social, and extracurricular activities (Fredricks et al., 2004), emotional engagement relates to affective connections with school (Fredricks et al., 2004; M. T. Wang et al., 2011), while cognitive engagement denotes mental investment in learning tasks, self-regulation, and value recognition (Fredricks et al., 2004). Notably, when examining classroom-specific contexts, scholars identified limitations in traditional three-dimensional models for explaining engagement behaviors (Pino-James et al., 2019). These studies emphasize functional aspects of student engagement in instructional settings: task persistence (e.g., learning duration and perseverance), classroom affect (e.g., emotional responses to academic work), and cognitive resource allocation (e.g., mental effort expended on tasks) (Pino-James et al., 2019). Distinct from general school engagement, classroom engagement has been demonstrated as critical for deep learning (Skinner et al., 2009). The new theoretical models (Skinner et al.’s (2008) Motivational Developmental Self-System Model; Reeve’s (2013) Four-Factor Model) have expanded on the original three dimensions to incorporate the dimensions of social engagement and agentic engagement. Social engagement emphasizes interaction quality in teacher-student and peer academic collaborations (Christenson et al., 2012; Fredricks et al., 2016; M. T. Wang et al., 2016), whereas agentic engagement reflects students’ proactive learning behaviors like questioning and idea-sharing (Reeve, 2013; Reeve & Tseng, 2011). Building on this progression, this study defines classroom engagement as multidimensional involvement across cognitive, affective, behavioral, and agentic domains, encompassing both task-focused mental exertion and positive instructional interactions with normative classroom conduct.

Previous studies have investigated classroom engagement through diverse methodological approaches. For instance, Amemiya et al. (2020) implemented a longitudinal diary study design, utilizing 15 consecutive daily surveys to examine how teachers’ disciplinary behaviors influenced adolescents’ subsequent-day behavioral engagement, while assessing the mediating role of student-teacher trust. Although empirical studies have identified engagement mechanisms in specific instructional contexts (Cinar et al., 2023; Cunha et al., 2023; Olivier et al., 2019), existing research predominantly adopts cross-sectional designs limited to single educational stages or specialized populations (Dykstra Steinbrenner & Watson, 2015; Hoang et al., 2019). Furthermore, systematic reviews have primarily focused on school-wide engagement rather than classroom-specific dynamics. Martins et al. (2022) systematically analyzed 102 articles to establish a comprehensive framework for school engagement research and provide empirical support for developmental trajectory studies. However, this broad institutional perspective fails to account for contextual variations in teaching environments, despite evidence demonstrating environmental influences on engagement patterns (Finn, 1989). In addition, current academic research on school engagement has yielded a wealth of findings, offering valuable theoretical frameworks and methodological insights that inform studies on classroom participation (Fredricks et al., 2004; M. T. Wang & Fredricks, 2014). A review of school engagement literature reveals that researchers’ efforts to expand conceptual dimensions and diversify methodological approaches have provided critical references for advancing classroom participation studies (Fredricks et al., 2004; Y. Li, 2011; Vargas-Madriz et al., 2024). However, existing research has inadequately emphasized the uniqueness and scholarly significance of classroom participation within specific instructional contexts. In summary, three critical gaps emerge: (1) a paucity of literature reviews specifically addressing classroom engagement, (2) limitations in quantitative methodologies for capturing developmental trends and contextual dynamics, and (3) insufficient translation of theoretical frameworks into practical pedagogical applications.

Bibliometric analysis serves as a robust methodological framework for examining academic journal dynamics, employing quantitative statistical approaches to systematically characterize scholarly literature within specific research domains (Wider et al., 2024). Developed by China scholar Dr. Chaomei Chen, CiteSpace constitutes a specialized visualization platform that enables (1) knowledge domain mapping through scientific literature measurement and analysis, (2) evolutionary pattern identification via co-occurrence networks (keywords, institutions, authors), and (3) research frontier visualization incorporating temporal trend analysis and conceptual relationship mapping. This computational tool transforms citation data into interactive knowledge landscapes, facilitating identification of pivotal literature, emerging research themes, and intellectual structure dynamics within disciplinary contexts (C. Chen et al., 2014; B. H. Li et al., 2017). As a result, the CiteSpace knowledge graph has gained popularity in a variety of scientific fields for drafting literature reviews because of its benefits.

In view of the dynamic characteristics of bibliometric analysis methods and CiteSpace, this study aims to apply bibliometric approaches to systematically organize and synthesize prior research on classroom engagement. Additionally, it will employ the CiteSpace (version 6.3.R3) software to analyze literature on classroom engagement retrieved from the Web of Science Core Collection spanning the past 49 years. The objective is to assist researchers in comprehending the trends and structure of this research domain, while providing references for in-depth exploration of current advancements, frontier dynamics, and future directions in this field. By utilizing these advanced analytical tools and leveraging data from the Web of Science Core Collection, this study will conduct a visualized analysis of literature on classroom engagement research from 1975 to 2023. This endeavor seeks to offer a comprehensive overview of research within this specific time frame, extract meaningful insights, and achieve the following objectives.

Q1: What distribution patterns characterize classroom engagement research across authorship, geographic regions, institutional affiliations, and journal outlets?

Q2: How have research hotspots and evolutionary trajectories developed in classroom engagement research over the past 49 years?

Q3: What emerging frontiers and conceptual innovations currently define the classroom engagement research domain?

## 2. Materials and Methods

### 2.1. Data Sources

Bibliometric analysis relies on objective and comprehensive scholarly databases. The Web of Science (WoS) Core Collection, one of the most established citation indexing systems, encompasses thousands of high-impact international journals across multidisciplinary domains, maintaining significant academic influence through its curated coverage of peer-reviewed literature (Ellegaard & Wallin, 2015). Compared to alternative databases, WoS provides superior citation metadata integrity and breadth of disciplinary coverage (M. Chen et al., 2022). As a premier digital repository for scientific literature, its rigorous journal selection criteria and citation tracking mechanisms have been extensively validated in bibliometric research (Jing et al., 2023). These attributes substantiate WoS as a rational and empirically sound data source for this investigation.

**Figure 1:**
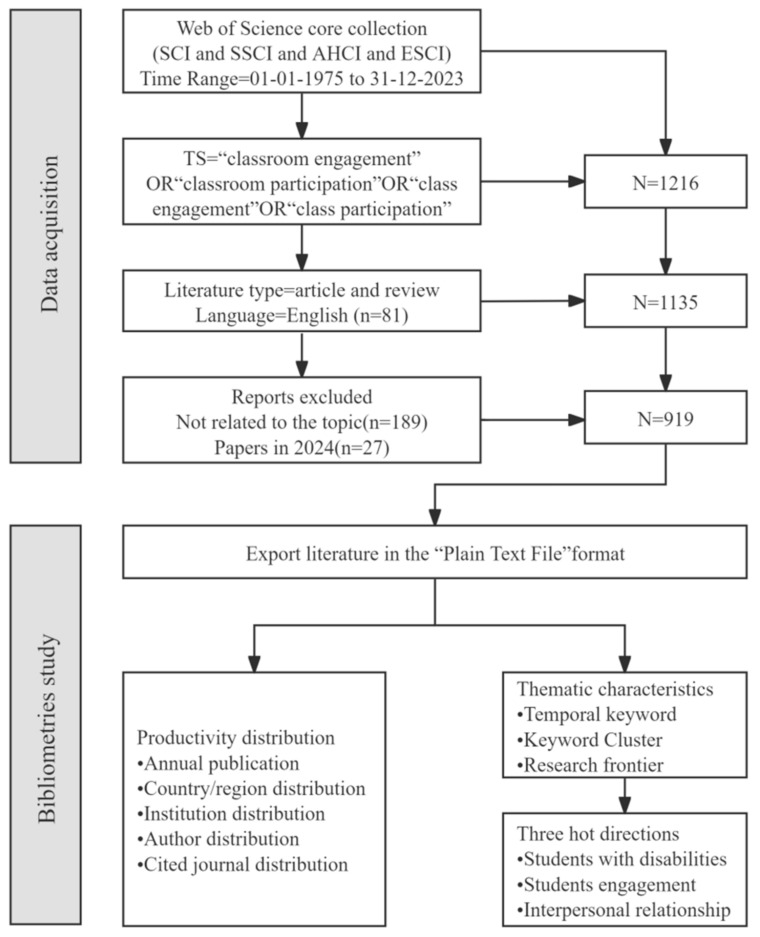
Structure of the Article.

The WoS database covers major international academic journals publishing papers on classroom engagement. Considering the concentration and quality of papers, this study only selects the Citation Index of Social Sciences in the WoS core database (Z. D. Li & Zhang, 2023). In the WoS Core Collection, articles related to classroom engagement were searched using the formula TS = (“classroom engagement” OR “class engagement” OR “classroom participation” OR “class participation”). The first article on classroom engagement was published in 1975. To avoid the impact of daily database updates on the search results, all searches were conducted on the same day, especially on 16 August 2024. Therefore, this study sets the literature publication time between 1975 and 2023 (data search ended on 31 December 2023). To obtain a wider range of high-quality research papers, indices like SCI-E, SSCI, AHCI, and ESCI were chosen, with literature types being Article and Review Article, and language set as English. After removing irrelevant and duplicate literature, 919 eligible papers were finally retrieved for further analysis. The article structure is shown in Figure 1.

### 2.2. Data Analysis

In this study, CiteSpace (version 6.3.R3) and Microsoft Excel (version 2019) were employed as analytical tools. First, the literature collected from the WoS Core Collection was downloaded in Plain Text File format and imported into CiteSpace (6.3.R3). The “Data” option within the software was utilized to perform format transformation and deduplication. Next, the parameters in CiteSpace were configured as follows: the time span was set from January 1975 to December 2023, with a time slice of 1 year. The analysis node types included publications, countries, journals, and keywords. Last, according to the requirements of software operation and analysis, to ensure the reliability and stability of nodes, the threshold was set to k = 25 and Top50, and the clustering label words were extracted according to the LLR loglikelihood algorithm.

In CiteSpace, the graph of scientific knowledge in a domain can be represented by various types of networks. In the generated knowledge graph, N represents the number of network nodes, E represents the number of connections, and Density represents the network density (Su et al., 2023). The connection line represents the cooperation or connection between the elements. In the country/region collaboration network, the connection line represents the collaboration between different countries/regions. In the keyword co-occurrence network, the connection line represents the co-occurrence relationship between different keywords (Tang et al., 2018). In the co-citation analysis graph, the size of nodes reflects the number of citations, the size of nodes in the co-citation network of journals reflects the number of citations of journals, and the lines between them reflect the intensity of co-citations. Cluster analysis refers to the analysis process of dividing a group of physical or abstract objects into multiple classes composed of similar objects (Alonso-Betanzos & Bolón-Canedo, 2018). Based on the cluster size and average year of publication, we can judge the hotspots in a field and their evolution (Bicheng et al., 2023). A burst indicates that the value of a variable changes greatly in a short time. Keywords with strong bursts indicate that researchers have discovered new research fields and viewpoints in a certain period, thus revealing the academic frontier of this certain period (S. Wang et al., 2023).

## 3. Results

### 3.1. Productivity Distribution

#### 3.1.1. Analysis of Annual Publication

**Figure 2:**
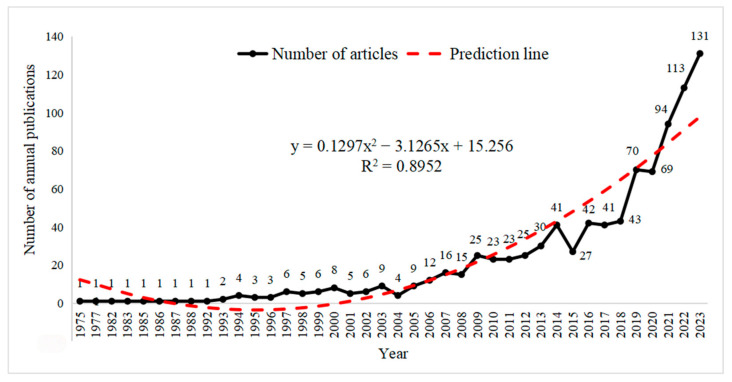
Number of Articles on Classroom Engagement from 1975 to 2023.

The annual publication output can reflect the developmental trends in a particular research field. Within the scope of the search, the annual publication output of studies on classroom engagement is shown in Figure 2. The development process of this field can be divided into three stages: the slow-growth stage (1975–2005), the rapid-advancement stage (2006–2018), and the sustained-growth stage (after 2019). It can be observed that 2005 is a turning point in terms of annual publication output. Before 2005, annual publications remained consistently low, fluctuating within single-digit figures, indicating limited academic attention to this topic. A significant surge occurred after 2005, with publications escalating from nine in 2005 to 43 by 2018. This stable and accelerated growth likely stemmed from two key factors: the widespread adoption of Fredricks et al.’s (2004) three-dimensional engagement model and educational policy reforms such as the U.S. No Child Left Behind Act, which prompted scholars to explore comprehensive educational quality metrics. These developments established classroom engagement as a prominent research focus. Since 2019, the field has entered a phase of sustained growth, achieving unprecedented publication volumes. This continued expansion demonstrates enduring scholarly interest, particularly driven by technological innovations and the global shift toward online education. Researchers increasingly emphasize how classroom performance critically influences students’ future development, aiming to enhance academic achievement and adaptability in complex learning environments.

#### 3.1.2. Country Distribution

**Figure 3:**
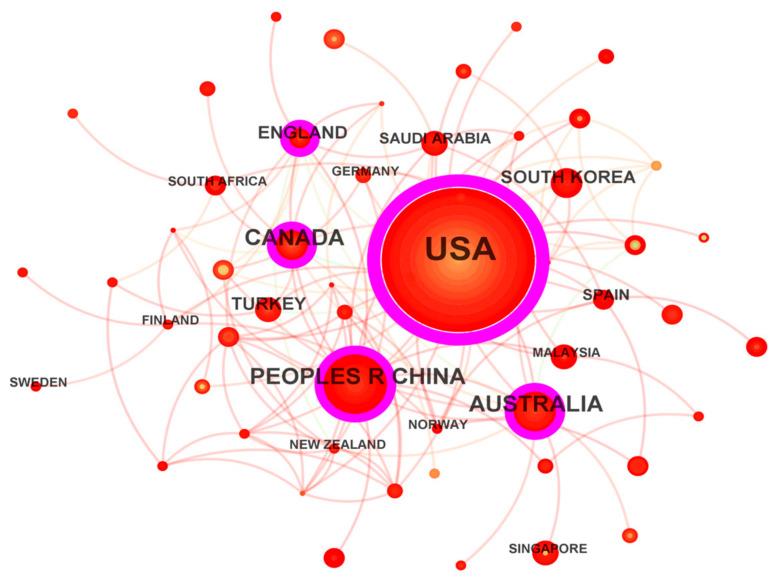
Network Knowledge Map of Country Collaboration.

Over the past 49 years, 74 countries have contributed to research on classroom engagement development and trends, forming a mature collaboration network. As shown in the knowledge map in Figure 3, the top three countries in terms of publication output are the United States (449 papers), China (74 papers), and Australia (63 papers). The U.S. leads with 449 papers, the highest number in the search area. It started earliest in classroom engagement research, collaborates with most countries, and has the highest centrality, indicating close research ties and significant innovative contributions, putting it at the forefront of classroom engagement studies. Greater national collaboration increases centrality, and multilateral research collaborations benefit the global development of the classroom engagement field. In terms of centrality, the top three countries are the U.S., China, and Australia. Nodes with a centrality greater than 0.10 are often key drivers of research changes. The U.S., China, Australia, Canada, and the UK all have centralities exceeding 0.10, showing their high innovation capacity and important role in classroom engagement development.

#### 3.1.3. Institutions Distribution

Table 1 lists the top 10 institutions ranked by publication output and centrality. Within the search scope, 398 institutions published studies on classroom engagement. The top 10 institutions accounted for 179 articles, accounting for 19.5% of all publications (see Table 1). In terms of volume, Arizona State University, Texas A&M University College Station, and the University of California System ranked highest, all based in the U.S., underscoring America’s key role in this field. For centrality, the University of California System, Arizona State University, and Beijing Normal University took the top three spots with centralities of 0.19, 0.13, and 0.13, respectively. Their high centrality scores indicate frequent occurrence on shortest paths within the network, reflecting their significant influence and importance in classroom engagement research.

#### 3.1.4. Author Distribution

The analysis of publication output reveals 615 authors contributing 919 publications. Table 2 identifies the top 10 authors ranked by publication volume and citation counts, highlighting key influencers in classroom engagement scholarship. These scholars’ works represent foundational contributions demonstrating sustained disciplinary impact. Upon observation, a distinct author collaboration group led by the prolific writer Fitzpatrick C emerged in the network. Her research primarily focuses on children’s executive functions, classroom engagement, and academic achievement (Côté-Lussier & Fitzpatrick, 2016; Fitzpatrick et al., 2014; Pagani et al., 2010). Future research in classroom engagement would benefit from larger-scale inter-institutional and interdisciplinary collaborations to further advance the field.

The most frequently cited author is Fredricks JA, whose research has advanced the conceptualization and measurement of classroom engagement. She proposed defining and measuring engagement across three dimensions: behavioral, cognitive, and emotional. Her work revealed that adolescents with higher levels of engagement exhibit stronger academic capabilities, closer connections to school, and more positive responses from teachers and parents (M. T. Wang & Fredricks, 2014). Encouraging adolescents to engage in classroom activities and thrive academically can reduce problem behaviors and yield long-term academic and educational benefits. Fredricks JA developed the Adolescent Engagement Scale, which serves as an indicator to assess student engagement. This tool helps teachers more effectively identify student issues and interact with them. The scale has been widely adopted by researchers worldwide. Skinner EA, the second most cited author, used empirical research to examine the impact of three dimensions of teacher behavior: engagement, structuring, and autonomy support on elementary students’ behavioral and emotional engagement throughout the school year (Skinner & Belmont, 1993). His work emphasized the importance of teacher-student relationships, particularly interpersonal interactions, in optimizing student motivation. Ladd GW has also played a significant role in research on student classroom engagement. His studies primarily focus on how the quality of teacher-student and peer relationships affects various aspects of school adaptation in preschool children. This research holds great significance for educators regarding teacher perceptions and teacher education (Ladd, 1990). The findings have been widely adopted by international researchers and have provided momentum for the development of classroom engagement research.

#### 3.1.5. Distribution of Cited Journals

**Figure 4:**
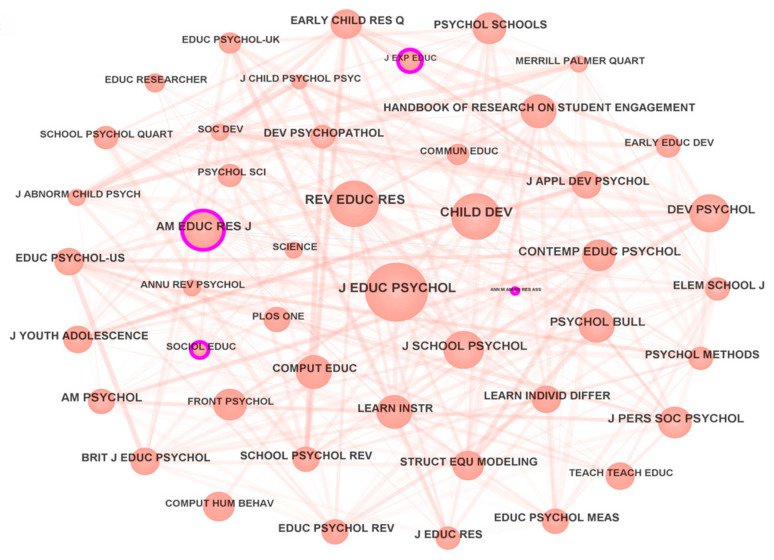
Network Knowledge Map of Cited Journals.

Journals are crucial for publishing high-quality papers. Two key metrics for assessing a journal’s influence in a field are its publication output and citation frequency. The co-citation network for journals in this study consists of 893 nodes and 3334 links, with a network density of 0.0084 (see Figure 4). Larger nodes indicate greater journal influence. The top three journals by citation count are *J EDUC PSYCHOL* (386 citations), *REV EDUC RES* (296 citations), and *CHILD DEV* (271 citations). *J EDUC PSYCHOL* is a highly influential journal in classroom engagement research. For example, Furrer, C. from Portland State Univ. published “Sense of relatedness as a factor in children’s academic engagement and performance” in it, which has been cited over 1346 times. To date, the journal has published 21 highly cited papers on classroom engagement. *REV EDUC RES*, a specialist educational research journal, ranks second. Finn, J. D. published “The ‘why’s’ of class size: Student behavior in small classes” in it in 2003, which has 181 citations. *CHILD DEV*, which focuses on child development, psychology, and education, rounds out the top three. In 1999, Ladd, G. W. published “Children’s social and scholastic lives in kindergarten: Related spheres of influence?” in this journal, which has over 722 citations.

### 3.2. Theme Characteristics

#### 3.2.1. Co-Occurrence of Keywords

**Figure 5:**
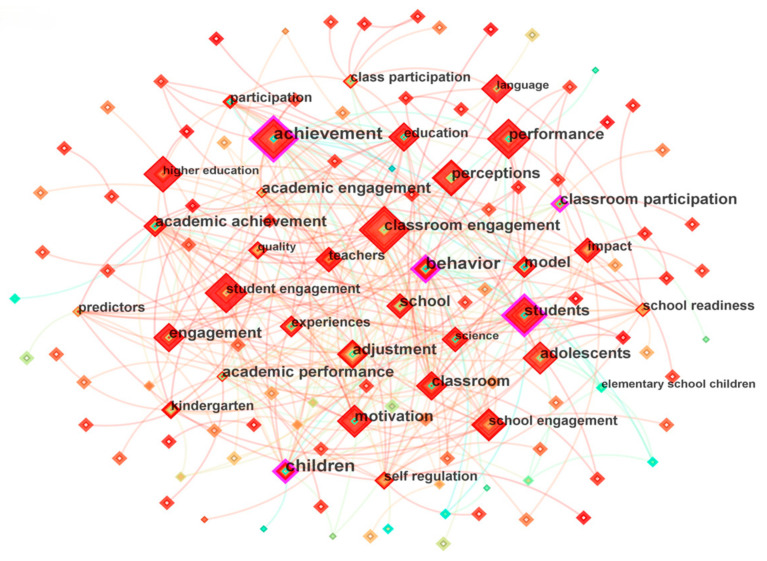
Co-occurrence Map of Keywords.

Keywords are essential for identifying the central themes and future developmental directions of publications. By examining keyword co-occurrence, researchers can gain insights into the current research and developmental trajectories within a specific field (Zhang et al., 2022). In this study, a total of 535 keywords were extracted from 919 articles related to classroom engagement, forming a network of 2287 links with a density of 0.016 (see Figure 5). Among these, 128 keywords with a frequency of five or more accounted for 23.93% of the total number of keywords. The most frequently occurring keyword was “classroom engagement” (131 times). Other prominent nodes included “achievement” (115 times), “student engagement” (89 times), “students” (86 times), “performance” (84 times), “engagement” (76 times), “motivation” (71 times), “behavior” (70 times), and “school” (67 times). In conjunction with the high-frequency keywords and relevant literature, it can be observed that research on classroom engagement primarily focuses on academic achievement, classroom performance, learning motivation, and behavioral engagement among adolescents in school settings. These variables are frequently examined as antecedents and outcomes in classroom engagement research.

#### 3.2.2. Analysis of Research Themes

**Figure 6:**
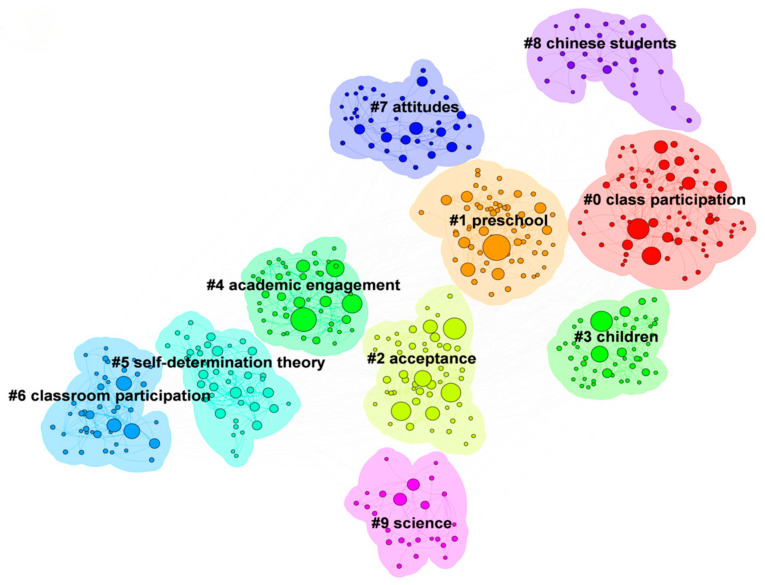
Cluster Map of Keywords.

Cluster analysis integrates related nodes and extracts keywords, which helps us intuitively understand the map’s content. Figure 6 displays the top 10 keyword clusters identified in the studies included. In this figure, the smaller the cluster ID, the more keywords the cluster contains. As shown in the Figure 6, two indicators are provided to evaluate the clustering effect: the modularity Q value and the average silhouette value (S value). The modularity Q value is 0.46, which is greater than the critical value of 0.30, indicating good clustering performance. The average silhouette value is 0.77, which is greater than 0.50, suggesting that the clustering results are significant and accurately represent the hotspots and themes in classroom engagement research (Pan & Yang, 2023). The unique clustering information constitutes the 10 keyword clusters that emerged (see Table 3). After summarizing and combining the research hotspots in this field using the cluster map and related indicators of cluster labels, it was found that the research hotspots exhibit “multi-dimensional diffusion.” The cluster labels extracted by CiteSpace can be broadly categorized into four core themes: students with disabilities (children, Chinese students), student engagement (class participation, preschool, academic engagement, classroom participation, attitudes), interpersonal relationships (acceptance, science), and self-determination theory.

#### 3.2.3. Research Frontier Analysis

**Figure 7:**
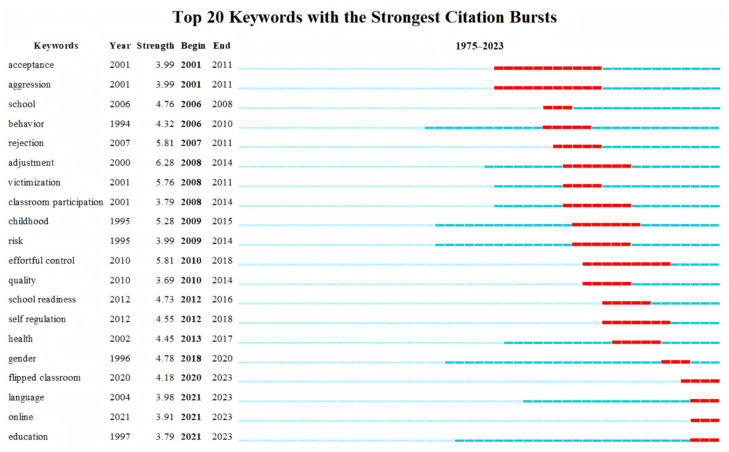
Top Twenty Keywords with the Strongest Emergence.

Keyword emergence refers to the phenomenon where the frequency of a keyword increases sharply over a period and receives significant attention. This can be used to assess the current hotspots in a research field, identify emerging frontiers, and reflect future developmental trends. In this study, we extracted emergent keywords for each year in the field of classroom engagement. The term “strength” represents the intensity of a keyword’s emergence, reflecting the research’s popularity. “Begin” and “End” indicate the first and last years of citation emergence for the corresponding keyword. As shown in Figure 7, the figure displays the top 20 keywords with the highest emergence rates from 1975 to 2023.

The research interests in this field are diverse, as shown in Figure 7. Between 2001 and 2011, the keywords “acceptance” and “aggression” received the most sustained attention, with scholars focusing on how peer rejection, peer acceptance, and aggression affect children’s school adaptation and classroom engagement. The keywords with the highest emergence strength are mostly concentrated between 2007 and 2015, and as of the end of 2023, the top three keywords in terms of emergence strength are “flipped classroom” (2020–2023), “language” (2021–2023), and “online” (2021–2023).

## 4. Discussion

### 4.1. Productivity Distribution

Over the past 49 years, classroom engagement has been widely discussed by researchers across disciplines, particularly in education and psychology. Through the citation-based knowledge mapping analysis conducted using CiteSpace, this study has gained a comprehensive understanding of the overarching framework of classroom engagement research. The findings reveal a consistent growth trend in research on this topic, with a particularly rapid acceleration post-2019. This surge may be attributed to the COVID-19 pandemic’s substantial impact on school education, prompting researchers to investigate changes in student engagement within online learning environments. The United States and European developed nations demonstrate the most extensive research scope in this field, maintaining central positions and significant academic influence. China ranks second in publication output, emerging as the most productive developing country. This phenomenon may stem from multiple factors. Firstly, developed countries possess well-established education systems that prioritize educational research, allocating substantial resources to explore teaching processes. Secondly, their cultural ethos inherently values academic innovation and evidence-based practices, driving continuous investigations into classroom engagement to enhance educational quality. Finally, mature research institutions and collaborative networks provide robust foundations for in-depth studies. China’s remarkable achievement as the second-largest contributor among developing nations reflects both rapid progress in education and growing research emphasis: increased investment in educational modernization and quality-oriented teaching reforms have motivated scholars to actively explore classroom engagement mechanisms. However, compared with the United States, China remains more limited in publication volume and international collaboration. Therefore, China needs to expand research efforts in this domain and broaden global partnerships to strengthen its academic impact.

The top 10 research institutions, predominantly universities, have played a pivotal role in advancing this field. Notably, 90% of these institutions are based in the United States, further underscoring the dominance of American academic entities in this research domain. In terms of author contributions, Fitzpatrick C is identified as the most prolific author, while Fredricks JA emerges as the most frequently cited scholar. Analyzing authors’ publication counts and citation frequencies helps identify representative researchers and core intellectual strengths within the field. Among cited journals, *J EDUC PSYCHOL* (*Journal of Educational Psychology*), which publishes studies on learning, instruction, and assessment in educational psychology, ranks highest. It is closely followed by *REV EDUC RES* (*Review of Educational Research*), a journal specializing in critical and integrative reviews of educational studies. The distribution analysis of highly cited journals provides researchers with guidance—to some extent—for locating academically significant studies in this discipline.

### 4.2. Development Trends and Frontier Hotspots

Keywords are extracted from the titles and subject matter of collected literature, not only reflecting the core perspectives of research topics but also providing insights into developmental trends within specific domains (Ji et al., 2023). Burst keyword analysis reveals that the strongest burst keywords from 2020 to 2023 primarily include “flipped classroom”, “language”, and “online”.

(1) “Flipped Classroom”: Technological innovation is challenging traditional teaching methods, and conducting teaching activities in new contexts holds significant importance. The flipped classroom is a novel instructional model that transforms the traditional teacher-centered classroom teaching approach, enabling students to adjust their learning pace and methods according to their own learning rhythms and styles (Clark, 2013). Moreover, the flipped classroom has been shown to lead to greater classroom engagement and better skill development, facilitate the cultivation of students’ autonomy and cooperative awareness, and enhance learning efficiency (Elmaadaway, 2018; He, 2020).

(2) “Language”: Language serves as the medium for classroom communication, and both verbal and written expression are fundamental ways for students to engage in classroom activities. Language courses are essential for students, and evidence suggests that students tend to be more engaged in language and literature courses than in mathematics courses (Sever et al., 2014). Furthermore, scholars have developed a second-language engagement scale, which has been widely used across various cultural contexts (Eerdemutu et al., 2024).

(3) “Online”: With the advancement of information technology, online classrooms and distance teaching have garnered increasing attention. Teachers can overcome the temporal and spatial constraints by conducting instructional activities online (M. Chen et al., 2022). This shift was particularly prominent during the COVID-19 pandemic, when traditional face-to-face teaching was transformed into online remote instruction and virtual guidance (Sadjadi, 2023). However, online teaching struggles to fully replicate the interactivity and supervision of in-person instruction, leading to differences in students’ classroom engagement and learning outcomes (Castro & George, 2021; Zhao & Akhter, 2023).

In forecasting future research trends, it is evident that blended learning models integrating online and offline education will remain a persistent focus in classroom engagement studies, driven by continuous technological advancements. Researchers will further explore optimization strategies for this model to enhance engagement and improve learning outcomes. Concurrently, investigations into classroom engagement within multilingual contexts are expected to gain momentum, addressing the demands of globalized educational exchanges and supporting the academic development of students from diverse cultural backgrounds. Furthermore, advancements in neuroscience and related fields may enable interdisciplinary integration with classroom engagement research. By uncovering the neural mechanisms underlying students’ engagement during instructional processes, such cross-disciplinary efforts could provide a scientific foundation for pedagogical practices and evidence-based educational methodologies.

### 4.3. Students with Disabilities and Classroom Engagement

The bibliometric findings of this study reveal that research on classroom engagement in special education exhibits a dispersed distribution in keyword clustering (Table 3), indicating that this area remains an emerging field. Educating children with disabilities or special needs in school settings has become a growing focus of attention and priority (Pijl, 2007). To better serve these children and design educational interventions that enhance their learning outcomes, researchers must investigate both individual and classroom-level factors influencing their educational experiences.

Studies have shown that children with disabilities in mainstream schools demonstrate lower classroom engagement and engagement in general school activities compared to their peers (Eriksson et al., 2007; Shevlin et al., 2002). Wendelborg and Tøssebro (2011) observed an overall decline in classroom engagement among children with disabilities from early to late primary school, with minimal changes for those with mild disabilities but significant decreases for those with moderate or severe disabilities. Additionally, children categorized with “intellectual”, “multiple”, or “other” disabilities participated less in classroom activities than those with physical disabilities. Selanikyo et al. (2017) demonstrated that collaborative consultation between Co-PID therapists and teachers, as an intervention model, improved classroom performance and engagement among students with intellectual and developmental disabilities, highlighting its effectiveness in supporting special education students. Clemons et al. (2016) provided empirical evidence that self-monitoring interventions enhance classroom engagement among high school students with disabilities. The acceptability of such interventions across diverse environments and teaching staff further positions them as advantageous for school professionals seeking efficient and adaptable solutions.

Despite these promising interventions, their long-term efficacy, adaptability to varying educational contexts, and scalability across cultural backgrounds require further validation. Looking ahead, as educational philosophies evolve and technological tools advance, personalized classroom engagement support strategies for students with disabilities are expected to diversify. Within the framework of inclusive education, fostering collaborative development in classroom engagement between students with disabilities and their neurotypical peers will emerge as a critical research direction.

### 4.4. Student Engagement

The keyword co-occurrence network (Figure 5) validates the multidimensionality of student engagement. The dense connections among keyword nodes such as “motivation”, “behavior”, “self-regulation”, and “self-determination theory” (Cluster 5) resonate with Reeve’s four-component conceptual framework. This framework comprises four primary dimensions: cognitive engagement, emotional engagement, behavioral engagement, and agentic engagement. Each dimension possesses distinct definitions and roles, collectively shaping individual development through their interconnected dynamics (Ladd & Dinella, 2009; Martins et al., 2022; Reeve & Lee, 2014; Reschly & Christenson, 2022).

(1) Cognitive Engagement

Cognitive engagement refers to students’ motivation, effort, and use of learning strategies in classroom settings (Wehlage & Smith, 1992). It is widely studied as a dichotomous process, exemplified by constructs such as deep/meaningful versus shallow cognitive engagement, deep versus surface processing, and engagement versus disengagement (Azevedo, 2015; Dinsmore & Alexander, 2012). Greene (2015) characterizes deep engagement as active application of prior knowledge and sophisticated strategies (e.g., monitoring and self-reflection) to generate complex knowledge structures, while shallow engagement involves deliberate yet mechanical strategies requiring limited cognitive deliberation. Students employ metacognitive strategies to plan, monitor, and evaluate cognition during task execution (Pintrich & De Groot, 1990). Additionally, they regulate effort expenditure through persistence or interference inhibition to sustain cognitive engagement (Pintrich & De Groot, 1990). Research indicates cognitive engagement constitutes fundamental processing operations that initiate or sustain student interactions with specific tasks, activities, and learning environments, inherent to all learning processes (Boekaerts, 2016).

(2) Emotional Engagement

Emotional engagement refers to students’ emotional reactions and attitudes in the classroom, including interest, enjoyment, and anxiety. Research on emotional engagement is related to students’ attitudes, as well as their interests and values (Epstein & McPartland, 1976; M. T. Wang & Eccles, 2012a). Studies have shown that both positive and negative emotional engagement are significantly associated with multiple indicators of academic and psychological functioning, both concurrently and prospectively (Y. Li & Lerner, 2011; M. T. Wang & Degol, 2014; M. T. Wang & Fredricks, 2014). Given the importance of school experiences in adolescents’ lives, increasing evidence suggests that students with higher levels of emotional engagement in the learning environment may have greater learning satisfaction and better mental health compared to those who experience anxiety, boredom, or disaffection (Carter et al., 2007; Luo et al., 2019; Van Ryzin et al., 2009). Students who are emotionally disengaged from their academic lives are also likely to become behaviorally and cognitively disengaged, leading to academic burnout and ultimately an increased risk of poor academic performance (Archambault et al., 2009; Hirschfield & Gasper, 2011; M. T. Wang et al., 2015).

(3) Behavioral Engagement

Behavioral engagement is described as students’ effort, attention, and persistence in initiating and performing learning activities (Skinner et al., 2009). Attention refers to children’s degree of focus on teacher-directed activities, reflecting levels of persistence, orientation, and task concentration (Georges et al., 2012). Children with poor attention may struggle to concentrate, avoid distractions, and lack perseverance (Miech et al., 2001). Research shows that high-achieving students often exhibit better compliance with instructions and task completion compared to their average-performing peers (Fredricks et al., 2004; McClelland et al., 2007). Higher levels of behavioral engagement are beneficial for adolescents’ academic outcomes (e.g., grades and performance), emotional outcomes (e.g., emotional regulation and conflict resolution skills), and social outcomes (e.g., social awareness and interpersonal skills) (Christenson et al., 2012). Conversely, early behavioral engagement problems can have long-term negative impacts, such as academic failure (Johnson et al., 2006) and internalizing and externalizing problem behaviors (Y. Li & Lerner, 2011). By providing social support, teachers and peers can influence the level of behavioral engagement over time (De Laet et al., 2015; M. T. Wang & Eccles, 2012b).

(4) Agentic Engagement

The concept of agentic engagement was initially proposed by Reeve and Tseng (2011), emphasizing students’ efforts to create a more motivationally supportive learning environment for themselves (Matos et al., 2018). They defined it as “the effort students make in response to the teaching they receive.” Specifically, students can proactively engage in the teaching process by expressing their preferences, asking questions, and letting teachers know what they need and what interests them. This helps to enhance their learning, secure the interpersonal support they need, and boost their task-related motivation (Reeve & Tseng, 2011; Reeve, 2013). This dimension emphasizes students’ autonomy and has drawn widespread scholarly attention since its introduction. Recent research has shown that students who resist the impulse to passively receive instruction and instead use their initiative to actively improve the quality of the instruction they receive experience a more supportive classroom environment and enhance their engagement and sense of achievement (Patall et al., 2019).

Current research on the multidimensional structure of student engagement has yielded notable insights, yet significant gaps persist in understanding the dynamic interactions between these dimensions and how comprehensive intervention strategies might simultaneously enhance active engagement across multiple facets. For instance, it remains unclear whether the relative importance of engagement dimensions or their interaction patterns vary across disciplines, instructional approaches, or educational stages questions current studies have yet to conclusively address. Furthermore, the interplay between non-cognitive factors (e.g., personality traits, social competencies) and multidimensional engagement structures requires deeper exploration. Future investigations will likely adopt interdisciplinary methodologies, integrating theories and techniques from psychology, education, neuroscience, and related fields to unravel the complexity of engagement mechanisms. Such efforts aim to develop targeted, evidence-based intervention strategies that holistically improve classroom engagement quality and learning outcomes.

### 4.5. Interpersonal Relationships and Classroom Engagement

The keywords covered in the keyword clustering data (Table 3), such as “acceptance”, “victimization”, “peer victimization”, “social acceptance”, “peer support”, and “peer acceptance”, further validate the profound impact of interpersonal relationships on classroom engagement. This indicates that the theme occupies a central position in related research fields and constitutes a key dimension for understanding classroom engagement phenomena. From an ecological perspective, engagement is not an individual characteristic but a relational one, emerging from social interactions within the classroom. It reflects the extent to which students are involved with activities or people in the classroom environment (Hofkens & Pianta, 2022). Previous research has shown that both individual characteristics and relational qualities in the classroom context play important roles in predicting students’ levels of classroom engagement (Hughes et al., 2008; Olivier et al., 2020; Shin & Chang, 2022). Teacher-student and peer relationships reflect students’ adaptive social functioning in the classroom. Children who establish supportive relationships with teachers and peers are likely to feel confident in their abilities and be more motivated to engage in classroom activities (Gest et al., 2005). Meta-analyses have confirmed the associations between positive (i.e., supportive and warm) and negative (i.e., conflictual) teacher-student relationships and student engagement (Roorda et al., 2011). The classroom peer ecology provides students with opportunities to participate in learning activities, practice social skills, receive constructive feedback, and share academic values and goals. All of these factors encourage students to become more involved in classroom learning activities (T. Li et al., 2024). Students’ social relationships with their classmates contribute to the development of their classroom engagement (Kindermann & Gest, 2018; Wentzel et al., 2020).

Current research lacks systematic empirical investigations into how to effectively establish and maintain positive interpersonal relationships across diverse educational stages and environments to enhance classroom engagement. This gap is particularly pronounced in atypical educational contexts or for student populations with special needs, such as understanding how teacher-student and peer relationships are cultivated and sustained in online learning environments, and how virtual social interactions might boost engagement among these students. As educational technology evolves and societal emphasis on personalized education grows, future studies will prioritize leveraging modern technological tools to optimize classroom relationships. This includes developing tailored social interaction strategies and digital platforms suited to varied educational scenarios and student demographics, thereby elevating the quality and efficacy of classroom engagement.

### 4.6. Self-Determination Theory

The fact that “self-determination theory” lies at the heart of cluster 5 (see Table 3) shows how crucial this theory is to the study of classroom participation. Self-Determination Theory (SDT, Ryan & Deci, 2000) emphasizes the relationships between social environments, self-needs, student engagement, and learning outcomes. The contextual variables refer to learners’ social environments, including teachers, parents, and peers. The self-related variables involve learners’ beliefs, values, and attitudes about meeting their needs for autonomy, competence, and relatedness. Student engagement, the third category, involves goal-directed behaviors, especially engagement in learning activities. The final component is outcomes, which in educational settings mainly refer to students’ cognitive development and academic achievement. According to SDT, high-quality motivation, engagement, and well-being come from meeting the basic psychological needs for autonomy (e.g., agency in one’s actions), competence (e.g., a sense of mastery), and relatedness (e.g., emotional connections with others). Effective educational environments are those that support rather than thwart these needs (Ryan & Deci, 2017). Thus, in educational processes, teachers can support high-quality motivation and engagement by using strategies that encourage students’ sense of autonomy and agency and avoiding controlling strategies that hinder students’ sense of autonomy (Jang et al., 2016; Patall et al., 2018; Patall & Zambrano, 2019).

## 5. Limitations and Future Research

Although this study provides a comprehensive and impartial analysis of classroom engagement publications using CiteSpace, several limitations should be acknowledged. First, the sample was exclusively drawn from the Web of Science (WoS) core collection to ensure data quality and completeness, potentially excluding relevant studies from other databases due to significant duplication across platforms. Additionally, differential access privileges among institutional subscriptions may introduce retrieval bias (Schwager & Schalk, 2022). Second, the restriction to English-language articles and reviews overlooks potential insights from working papers, conference proceedings, and other publication types. Third, while bibliometric analysis effectively mitigates bias, it cannot substitute thorough content analysis but merely complements it. Notably, CiteSpace’s knowledge mapping analysis demonstrates limitations in processing unstructured data and conducting fine-grained collaboration network analyses despite its strength in capturing evolutionary dynamics. Future research could enhance robustness by integrating VOSviewer’s community detection or Bibliometrix’s multidimensional metrics. Finally, although professional software ensures objective quantitative outcomes, data interpretation inevitably involves subjective elements. Subsequent studies should expand literature sources, incorporate expert consultations for balanced perspectives, and implement methodological safeguards against analytical subjectivity.

## 6. Practical Implications

While many prior studies have examined individual or localized facets of classroom engagement, this review is the first to seamlessly integrate a wide array of elements. It covers the multidimensional aspects of classroom engagement, including cognitive, emotional, behavioral, and agentic dimensions. It delves into interpersonal dynamics, looking at teacher-student and peer relationships. This review also explores the participation of special populations, such as students with disabilities, and traces developmental trends and cutting-edge topics like online education and flipped classrooms. Furthermore, it examines relevant theoretical frameworks, such as self-determination theory. This review breaks down disciplinary barriers and adopts a cross-disciplinary viewpoint to explore the intricate mechanisms of classroom engagement in depth. It provides an in-depth examination of the dynamic interplay between educational technology integration and multidimensional engagement in online settings. By doing so, it offers researchers in education, psychology, and other related fields a holistic and systematic framework for studying classroom engagement, effectively bridging the gap left by prior fragmented research.

Beyond its academic contributions, this comprehensive framework carries significant practical implications. It offers robust theoretical foundations and concrete implementation strategies for educational policymaking, classroom practice optimization, and teacher professional development. By facilitating the transformation of theoretical insights into actionable educational practices, it ultimately enhances educational quality and elevates student learning experiences through improved classroom engagement.

(1) Educational Policy

Policymakers should prioritize creating a macro-environment conducive to student classroom engagement. At the policy level, schools actively implementing pedagogical innovations could receive targeted resource support, such as funding and teacher training. Additionally, reforming the assessment system is critical. Traditional exam-centric evaluations may suppress students’ willingness to engage in classrooms. Educational policies should guide the establishment of a diversified assessment system that incorporates metrics like the frequency of student questions, contributions in group discussions, and performance in classroom presentations. This institutional shift will formally recognize and safeguard the importance of classroom participation.

(2) Classroom Practices

Research highlights themes such as students with disabilities and interpersonal dynamics in classroom engagement, suggesting that teachers should integrate differentiated strategies into instructional design. For example, multi-sensory engagement activities could be developed for special education students, while group collaboration can enhance peer interactions. As flipped classrooms remain a trending topic, teachers are advised to combine online resources (e.g., prerecorded videos) with in-class interactive sessions to foster active participation. In language classes, replacing one-way lectures with output-driven approaches (e.g., debates, speeches) can boost students’ expressive motivation. For online education, high-frequency interactive elements (e.g., real-time polls, virtual breakout discussions) should be designed to counteract reduced engagement caused by screen-mediated isolation.

(3) Teacher Development

Research indicates that teacher-student relationships and peer interactions significantly influence student engagement. To strengthen social connections, it is recommended to regularly conduct class-building activities such as role-playing and project-based learning. Teachers need to master techniques for enhancing student participation, such as designing moderately challenging questions that stimulate interest and critical thinking. Additionally, teachers should organize effective group activities, including appropriate grouping, role assignment, and facilitated discussions. Furthermore, teachers must focus on cultivating students’ learning capabilities and attitudes, as active classroom engagement enables students not only to acquire knowledge but also to develop crucial skills like critical thinking, collaboration, and communication. Teachers should recognize that their responsibility extends beyond knowledge transmission to helping students learn how to learn, thereby laying the foundation for lifelong development.

## 7. Conclusions

This study demonstrates that research on classroom engagement has generally increased over the past 48 years. Arizona State University, Texas A&M University College Station, and the University of California System are the most prolific institutions, consolidating the United States’ dominant position in this field. The close connections between different countries and institutions indicate that researchers value cross-national and cross-institutional communication and collaboration, which helps enhance the quality and impact of research. Fitzpatrick C is the most representative prolific author, while Fredricks JA is the most frequently cited author. The primary journals in the field of classroom engagement include *J EDUC PSYCHOL*, *REV EDUC RES*, and *CHILD DEV*. In recent years, keywords such as “flipped classroom”, “language”, and “education” have gained significant popularity in academic literature, attracting considerable attention. Additionally, the main topics explored in this field involve three directions: students with disabilities, interpersonal relationships, and student engagement.

## Figures and Tables

**Table 1:** Top 10 institutions in terms of publication output and centrality.

Institutions	Count	Institutions	Centrality
Arizona State University	39	University of California System	0.19
Texas A&M University College Station	24	Arizona State University	0.13
University of California System	20	Beijing Normal University	0.13
Université de Montréal	19	University of Massachusetts System	0.13
University System of Ohio	17	University of Texas System	0.12
California State University System	15	University of Auckland	0.08
Pennsylvania Commonwealth System of Higher Education (PCSHE)	13	University of Massachusetts Boston	0.08
University of North Carolina	12	Cornell University	0.07
State University System of Florida	10	California Polytechnic State University	0.06
University of Virginia	10	University of Kansas	0.06

**Table 2:** Author Distribution.

Authors	Count	Cited Authors	Cited Count
Fitzpatrick C	15	Fredricks JA	182
Pagani LS	7	Skinner EA	133
Hwang GJ	5	Ladd GW	108
Downer JT	5	Finn JD	95
Archambault I	5	Pianta RC	80
Hughes JN	5	Muthen LK	77
Buhs ES	5	Buhs ES	74
Valiente C	4	Hu LT	74
Reeve J	4	Ryan RM	72
Almqvist L	4	Reeve J	69

**Table 3:** Keyword Cluster Information.

Cluster ID	Size	Silhouette	Mean (Year)	(Label) LLR
0	69	0.77	2009	class participation; active learning; east Asian students; prior knowledge; faculty
1	64	0.71	2013	preschool; school readiness; classroom engagement; task engagement; self-regulation
2	51	0.76	2006	acceptance; victimization; well-being; peer victimization; social acceptance
3	47	0.85	2000	children; meta-analysis; prevalence; disability; special education
4	45	0.78	2006	academic engagement; behavioral engagement; behavior; stress; school transition
5	44	0.77	2012	self-determination theory; need satisfaction; relational goals; teacher self-efficacy; achievement goals
6	42	0.75	2006	classroom participation; instruction; elementary students; peer acceptance; silence
7	41	0.69	2012	attitudes; knowledge; transition; engagement; middle school
8	27	0.80	2020	Chinese students; pandemic; hard of hearing; deaf; online learning
9	26	0.82	2008	science; women; stereotype threat; stem; peer support

## Data Availability

Not applicable.
